# The effectiveness and efficiency of asymptomatic SARS-CoV-2 testing strategies for patient and healthcare workers within acute NHS hospitals during an omicron-like period

**DOI:** 10.1186/s12879-023-08948-9

**Published:** 2024-01-08

**Authors:** Stephanie Evans, Nichola R. Naylor, Tom Fowler, Susan Hopkins, Julie Robotham

**Affiliations:** 1 Clinical and Public Health Group, UK Health Security Agency, London, UK; 2 Data, Analytics and Surveillance Group, UK Health Security Agency, London, UK; 3 William Harvey Research Institute, Queen Mary University of London, London, UK; 4 NIHR Health Protection Research Unit in Healthcare Associated Infections and Antimicrobial Resistance, University of Oxford in partnership with the UK Health Security Agency, Oxford, UK

**Keywords:** Modelling, COVID-19, Lateral flow testing

## Abstract

**Background:**

Asymptomatic SARS-CoV-2 testing of hospitalised patients began in April-2020, with twice weekly healthcare worker (HCW) testing introduced in November-2020. Guidance recommending asymptomatic testing was withdrawn in August-2022. Assessing the impact of this decision from data alone is challenging due to concurrent changes in infection prevention and control practices, community transmission rates, and a reduction in ascertainment rate from reduced testing. Computational modelling is an effective tool for estimating the impact of this change.

**Methods:**

Using a computational model of SARS-CoV-2 transmission in an English hospital we estimate the effectiveness of several asymptomatic testing strategies, namely; (1) Symptomatic testing of patients and HCWs, (2) testing of all patients on admission with/without repeat testing on days 3 and 5–7, and (3) symptomatic testing plus twice weekly asymptomatic HCW testing with 70% compliance. We estimate the number of patient and HCW infections, HCW absences, number of tests, and tests per case averted or absence avoided, with differing community prevalence rates over a 12-week period.

**Results:**

Testing asymptomatic patients on admission reduces the rate of nosocomial SARS-CoV-2 infection by 8.1–21.5%. Additional testing at days 3 and 5–7 post admission does not significantly reduce infection rates. Twice weekly asymptomatic HCW testing can reduce the proportion of HCWs infected by 1.0-4.4% and monthly absences by 0.4–0.8%. Testing asymptomatic patients repeatedly requires up to 5.5 million patient tests over the period, and twice weekly asymptomatic HCW testing increases the total tests to almost 30 million. The most efficient patient testing strategy (in terms of tests required to prevent a single patient infection) was testing asymptomatic patients on admission across all prevalence levels. The least efficient was repeated testing of patients with twice weekly asymptomatic HCW testing in a low prevalence scenario, and in all other prevalence levels symptomatic patient testing with regular HCW testing was least efficient.

**Conclusions:**

Testing patients on admission can reduce the rate of nosocomial SARS-CoV-2 infection but there is little benefit of additional post-admission testing. Asymptomatic HCW testing has little incremental benefit for reducing patient cases at low prevalence but has a potential role at higher prevalence or with low community transmission. A full health-economic evaluation is required to determine the cost-effectiveness of these strategies.

**Supplementary Information:**

The online version contains supplementary material available at 10.1186/s12879-023-08948-9.

## Background

COVID-19 is a respiratory disease caused by the virus Severe Acute Respiratory Syndrome Coronavirus-2 (SARS-CoV-2). Since the start of the COVID-19 pandemic there has been evidence of nosocomial transmission of SARS-CoV-2 affecting both patients and HCWs [1–4], and it has been estimated that over 15,500 inpatients developed a nosocomial SARS-CoV-2 infection in England during the first wave alone [1]. The frequency of asymptomatic or paucisymptomatic infection has changed throughout the pandemic, but for the Omicron variant 20–60% of all cases are thought to be pauci or asymptomatic [5, 6], and 50–60% of hospital admissions that test positive for SARS-CoV-2 are in patients admitted for non-COVID-19 reasons [7]. Despite high levels of uncertainty there is evidence that the secondary attack among contacts of asymptomatic and presymptomatic cases is lower than among contacts of symptomatic cases [8, 9], however asymptomatic transmission is documented in cluster investigations [10].

Testing of asymptomatic patients on admission to hospital was standard practice from 27-April-2020 [11], but due to the turnaround time of PCR tests, patients were often cohorted by symptom status while awaiting test results [12]. Following the introduction of rapid testing (with lateral flow devices, LFDs) obtaining patient test results became quicker, and healthcare workers (HCWs) also underwent regular twice-weekly asymptomatic testing following the introduction of LFDs in November 2020 [13] at significant cost. Modelling studies have shown that twice-weekly asymptomatic testing of healthcare workers can detect 30% of pre-symptomatic cases and 75% of asymptomatic cases within 7 days of exposure [14] and that regular testing can reduce transmission to HCWs [15], however these studies do not consider the impact of detecting HCW infections on prevention of onward transmission to patients or staff absences, and no cost-effectiveness studies of asymptomatic testing protocols exist. Asymptomatic testing of patients and HCWs was withdrawn on 31 August 2022 [16]. Due to other changes in infection prevention and control (IPC) practices, the rate of community transmission [17] and the potential change in case ascertainment rate resulting from unidentified asymptomatic cases it is not possible to directly determine the impact of this policy change on transmission rates from currently accessible data sources alone. We present a computational modelling study estimating the impact of stopping asymptomatic testing on patient and HCW infections.

Building on a previously published computational model of SARS-CoV-2 transmission in NHS hospitals [18], we estimate the benefits and efficiency of different asymptomatic testing protocols for patients and HCWs in terms of infection incidence, HCW absences and volume of tests required. In the absence of detailed studies around additional health-related costs (e.g. attributable length of stay and mortality or quality of life years lost) resulting from omicron infection, evaluation of test per infection or absence prevented only can provide an indicative measure of efficiency of a health policy to inform decision-making.

In this modelling study, the alternative testing strategies are assessed over a 12-week period with Omicron as the dominant variant. We considered four different levels of prevalence ranging from low (0.5-1%) to very high (4–8%) and quantified the effectiveness and efficiency of each testing strategy in each setting. Understanding the efficiency of different asymptomatic testing protocols, including frequency of testing and who is being targeted (i.e. patients and/or HCWs) in terms of tests needed per patient and HCW infection averted, is important for resource allocation decisions to maximise the health gains from set budgets. We consider only the impact of testing for preventing nosocomial transmission in a hospital setting and therefore discharge testing (e.g. for return to a long-term care facility) is not included in this analysis. Similarly we do not consider the benefit of testing for clinical care.

## Methods

### Individual-based model

#### Model setup and parameterisation

We extended a previously developed individual-based, dynamic model of within-hospital transmission [16] to include effects of vaccine waning and immune-escape of the omicron variant. The model simulates transmission within and between patient and HCW populations in a typical English hospital (a single NHS hospital with 1000 beds and 8000 HCWs), and is then scaled to represent all NHS acute hospitals. The simulation period is 12 weeks with omicron as the dominant variant to enable a robust comparison between simulated testing strategies. A full model description and parameter table is given in Supplementary File 1, with the key features highlighted below.

In the model, simulated patients are representative of any patient admitted to an acute NHS hospital in England and can be admitted as Susceptible (not infected), Exposed (infected but not yet detectable or infectious), Infected (either asymptomatic, pre-symptomatic, or symptomatic, these patients are detectable and infectious), or Recovered (previously infected with SARS-CoV-2 so have some level of immunity to reinfection). If a susceptible patient becomes symptomatically infected they have an additional length of stay of 1.5 days added to their previously generated discharge date [19], however patients that are Exposed, Asymptomatic, or Presymptomatic do not have any additional length of stay added. Exposed individuals (patients and HCWs) transition to the next infectious state according to their Ct values that are calculated using the Kissler model [20]. An individual is assigned to a symptomatic or asymptomatic pathway on exposure, with a Ct value of 30 used for the threshold for infectiousness in either case [21] and symptom onset time coinciding with the maximum Ct value as described in previous models of viral trajectories [22] and parameterised using data from the literature [20, 23, 24].

The individual-based model has previously been calibrated to infection data from Secondary Uses Service (SUS) assuming 30% (21–40%) of nosocomial infections will be detected before discharge [25] and HCW infection data from the SARS-CoV-2 Immunity and ReInfection EvaluatioN study (Supplementary File 1).

**Fig. 1:**
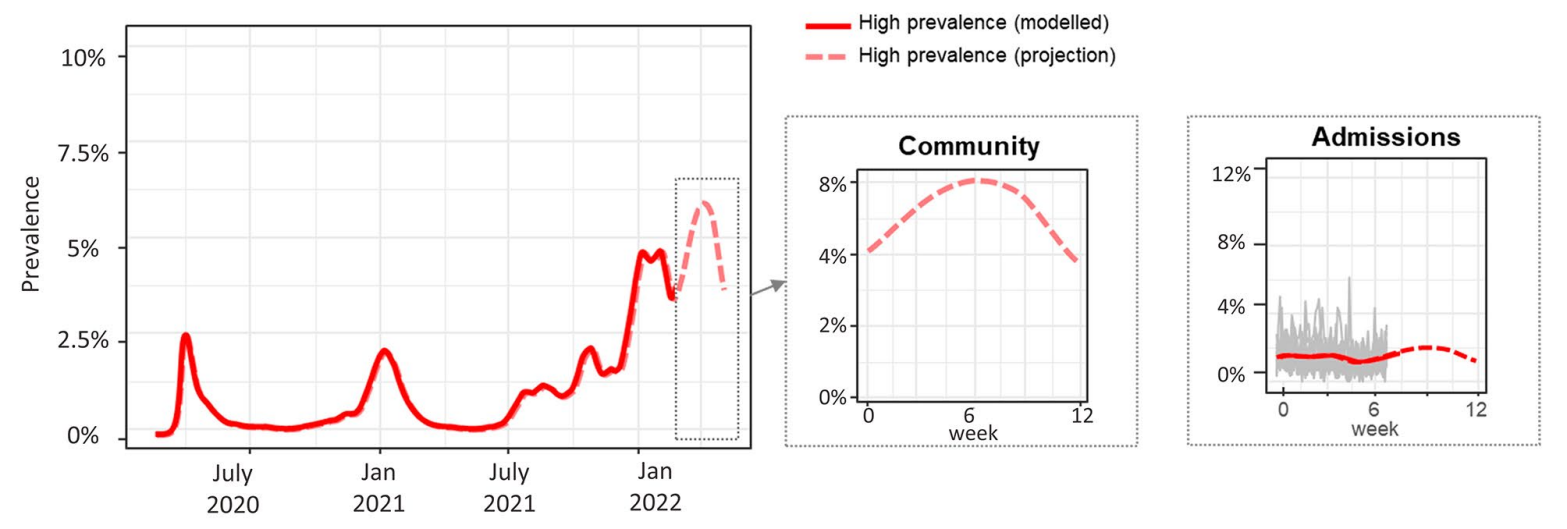
Modelled (solid lines) and predicted (dashed lines) community prevalence (**A**) and hospital admissions (**B**). Grey lines = variation in known admissions rates between trusts from NHSE Situation Report dataset

The model was simulated with hypothetical SARS-CoV-2 community prevalence levels of low (0.5-1%), medium (1–2%), high (2–4%) and very high (4–8%). Admission rates in each prevalence scenario were estimated using the relationship between modelled community prevalence rates from the PHE-Cambridge Real Time Model [26] and NHS Situation Report admissions, as demonstrated in Fig. 1. The proportion of admissions that were asymptomatic was taken to be 50% [7]. The probability of a patient being exposed (not yet detectable) on admission was drawn from projected community prevalence rates. We assume that at baseline 50% of HCWs and 30% of patients had previously been infected with non-Omicron SARS-CoV-2 variants and 90% of HCWs and 85% of patients over 50 had received a booster vaccine (set according to simulated age on admission using National Immunisation Management System data up to March 2022), and were therefore less likely to be infected with Omicron. Vaccine efficacy for individuals with and without prior infection was taken from published English estimates [27]. Protection from previous infection is fixed over the simulation period, but vaccine efficacy wanes over time.

#### Simulation of testing strategies

We considered 6 testing strategies consisting of all unique combinations of the three patient and two HCW testing options (Table 1) covering the range of asymptomatic testing strategies in place over the course of the pandemic in England. Symptomatic testing occurred in all testing strategies and the base-case was taken to be a scenario with no asymptomatic testing of patients or HCWs. In all scenarios, if a HCW receives a positive test result they isolate for 7 days before retesting daily (with an LFD test) and return to work when the test result is negative. If a patient receives a positive test result on admission, then they are cohorted in wards and bays with other SARS-CoV-2 positive patients, and if a patient receives a positive result while on a ward, then the ward is closed to new patients until no known SARS-CoV-2 positive cases are present before being reopened to new, non-infected patients. Patients with a positive test that remain asymptomatic do not have an extended length of stay. Patients that are discharged while incubating an infection have a probability of being readmitted if they become symptomatic after discharge. Patients that are in hospital when symptoms develop draw a COVID-19 specific length of stay from the admission duration distribution of COVID-19 patients (based on age and gender), and are then assigned a length of stay that is the maximum of possibilities (i.e. maximum value of their previously allocated length of stay and the newly drawn disease length of stay (so as to avoid shortening the stay of patients that would have stayed a longer time for their primary admission reason). As the model is transmission dynamic, with transmission within and between patient and HCW populations, each combination of the unique patient and HCW testing strategies had to be explicitly explored - as the strategy implemented for patients would impact HCW infections and vice versa.

**Table 1:** Patient and Healthcare Worker (HCW) testing strategies

*Patient testing strategies*	
Sympt	Symptomatic patients are tested within 2 days of developing symptoms and ward is closed to non-COVID admissions if possible
Adm	All admissions are tested as well as symptomatic patients, and positives are cohorted in the same bay/ward.
d5 + d3	All admissions and symptomatic patients are tested and appropriately cohorted and there is an additional retest on days 3 and 5 of an inpatient stay
*HCW testing strategies*	
None	Symptomatic HCWs test and isolate if they test positive. For calculating numbers of tests it is assumed that these HCWs take additional tests on days 5–10 of their isolation.
Asymp HCW testing	In addition to the symptomatic testing described in the “None” scenario, 70% of HCWs undergo twice weekly asymptomatic testing and isolate if positive, the remaining 30% do not participate in asymptomatic testing.

Each testing strategy was simulated using 20 unique previously calibrated parameter sets to capture uncertainty in the contribution of different pathways of nosocomial transmission (parameter uncertainty, 120 combinations in total), and each parameter set was simulated 5 times to explore stochastic uncertainty. The modelling framework is described schematically in Supplementary Fig. 1.

#### Scaling to national level data

To scale patient results from a simulated individual hospital to a national-level, data on all English hospital admission episodes from October 1st 2021 to December 31st 2021 were obtained from SUS, from which patient hospital spells were formed from contiguous in-patient episodes at a single hospital trust. These data were aggregated by age and gender, and combined with modelled estimates of the proportion of patients of the same age and gender that developed a nosocomial SARS-CoV-2 in the simulation per week.

HCW infection rates were scaled to a national level assuming that there are 700,000 front-line HCWs and combining this number with the proportion of HCWs infected in the simulated single hospital per week.

Total test and infection numbers were compared to respective values in the base-case “symptomatic only” testing scenario. Efficiency was defined as infections prevented per test implemented.

Model structure, assumptions and parameter inputs, as well as preliminary outputs were informed by consultation with clinicians and experts in the areas of infection prevention and control and healthcare-associated infections.

### Sensitivity analysis

A sensitivity analysis was performed by varying parameters related to transmission rates, asymptomatic probabilities, and vaccine efficacy. 1000 Latin-hypercube sampled parameter sets were generated within the ranges in Supplementary Table 1 using the *R* package *spartan* [28]. Simulations were executed for 12 weeks under a medium prevalence setting with Omicron as the dominant variant both with and without asymptomatic testing of patients and HCWs. Partial-rank-correlation coefficients were then calculated between each parameter and the total number of patient infections, the number of patient infections averted by asymptomatic testing, the total number of HCW infections, and the number of HCW infections averted by asymptomatic testing over the simulation period also using the *spartan R* package [28].

### Computational platform

The IBM is constructed in Java under Java SE 11 using the Multi-Agent Simulation Of Neighbourhoods (MASON) framework [29].

## Results

### Patient and HCW infections & HCW absences prevented by asymptomatic testing

#### Asymptomatic patient testing

**Fig. 2:**
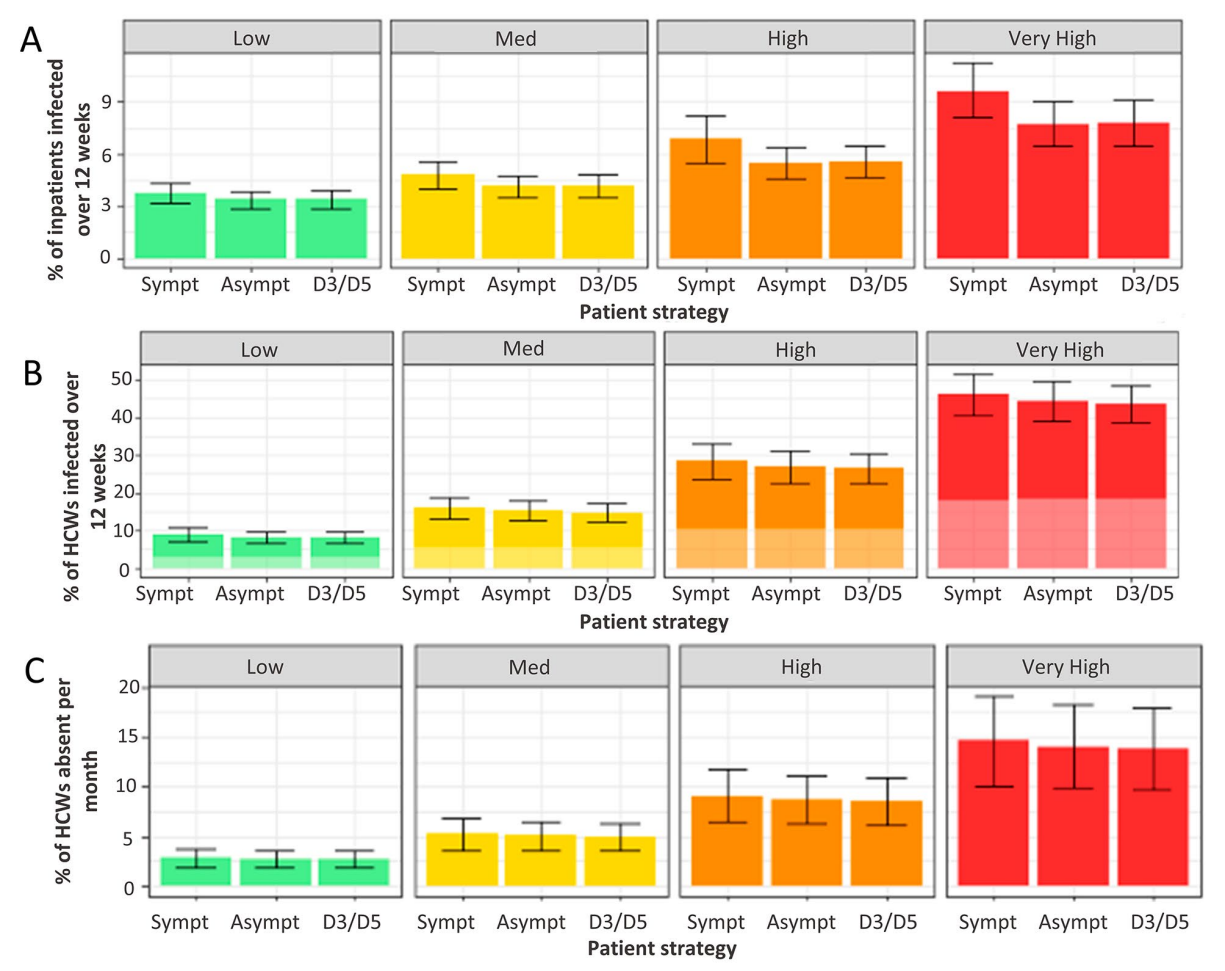
Impact of asymptomatic patient testing on infections in patients and HCW populations. (**A**) Percentage of patients infected nosocomially over 12 weeks. (**B**) Percentage of HCWs infected nosocomially (dark bars) and in the community (pale bars) over 12 weeks. (**C**) Percentage of HCWs absent with a detected or SARS-CoV-2 infection per month. In all scenarios HCWs are tested twice weekly with 70% compliance. Bars: median, Error bars: IQR

Over a 12-week period, where patients are tested on admission and again at days 3 and 5–7 post-admission, and upon developing symptoms, and HCWs are tested twice weekly with 70% compliance, 3.4% (median, IQR 3.2–3.6%) of patients and 3.0% (2.0-4.4%) of HCWs are nosocomially infected with SARS-CoV-2 in a low prevalence scenario (Fig. 2, Supplementary Table 2). This equates to 136,000 (128,000–144,000) patients and 20,900 (14,200 − 30,700) HCWs across NHS acute hospitals. Over the same time period when the community prevalence was set to be very high, 8.0% of patients (7.0-8.8%) and 16.4% (11.4–22.1%) of HCWs are nosocomially infected, equating to 323,000 (284,000–354,000) patients and 115,000 (80,000–155,00) HCWs. The monthly COVID-19 absence rates for HCWs increased from 2.4% (2, 3.2%) in the lowest prevalence scenario to 14% (11.2, 16.4%) in the highest prevalence scenario.

Removing all asymptomatic testing from the patient population while continuing to test asymptomatic HCWs twice a week results in an additional 0.5% (0-0.8%) of patients and 0.8% (-0.5-2.2%) of HCWs becoming infected. In the highest prevalence scenario,1.9% (0.7–2.9%) of patients and 2.6% (-1.9-7.2%) of HCWs are infected. This is equal to approximately 19,000 (1,200 − 31,000) patients and 5,500 (-3,000–15,000) HCWs in the lowest prevalence scenario and 80,000 (30,000–120,000) patients and 18,000 (-13,000–50,000) HCWs when the community prevalence is very high. Removing patient testing did not increase monthly absence rates in the lowest prevalence scenario but had a small impact in the very high prevalence scenario, increasing absence rates in HCWs by 0.4% (-0.8-2.4%).

Testing patients at days 3 and 5–7 post admission did not reduce the nosocomial infection rate in either the patient or the HCW population. Testing patients asymptomatically did not significantly impact the overall proportion of beds occupied by SARS-CoV-2 positive patients (Figure S2).

#### Asymptomatic HCW testing

**Fig. 3:**
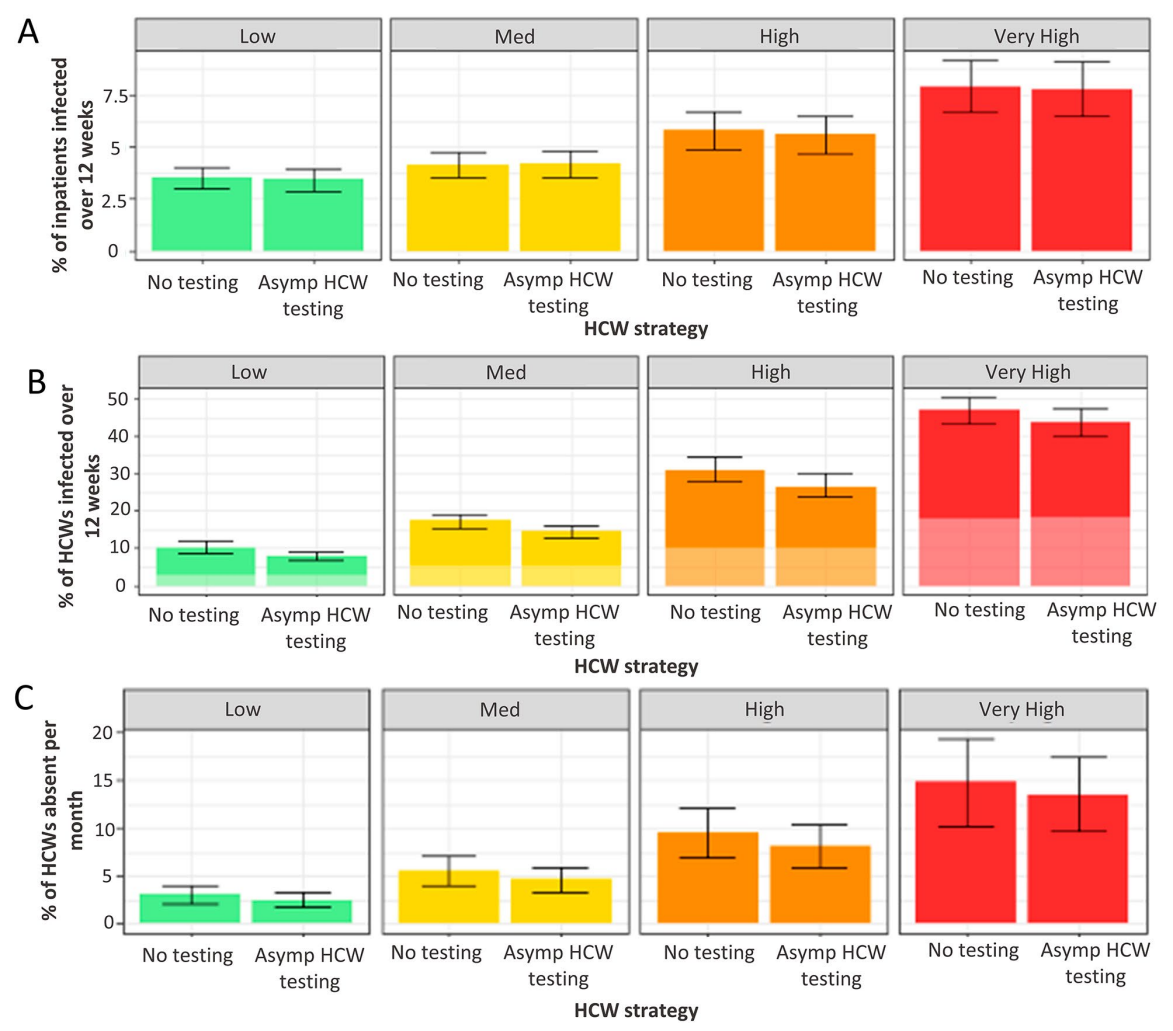
Impact of twice weekly asymptomatic HCW testing on infections in patients and HCW populations. (**A**) Percentage of patients infected nosocomially over 12 weeks. (**B**) Percentage of HCWs infected nosocomially (dark bars) and in the community (pale bars) over 12 weeks. (**C**) Percentage of HCWs absent with a detected or SARS-CoV-2 infection per month. In both scenarios all patients are tested on admission and at days 3 and 5–7. Bars: median, Error bars: IQR

Removing asymptomatic testing of HCWs while continuing to test patients on admission does not affect patient infections but has a significant impact on HCW infection (Fig. 3, Supplementary Table 3). In the lowest prevalence scenario, an additional 2.3% (1.1–3.6%) of HCWs are infected over the 12-week simulation period, this is 16,000 (8,000-250,000) individuals. In the highest prevalence scenario an additional 3.5% (-0.7-8.1%) of HCWs are infected when asymptomatic testing of HCWs is removed, 25,000 (-5000-55,000) total. Monthly HCW absence rates increase by 0.4% (0-1.2%) in a low prevalence scenario and 0.3% (-0.1-0.7%) in the highest prevalence scenario. Asymptomatic HCW testing did not cause a significant change in staff absent at any one time (Figure S3).

### Efficiency of testing strategies

**Fig. 4:**
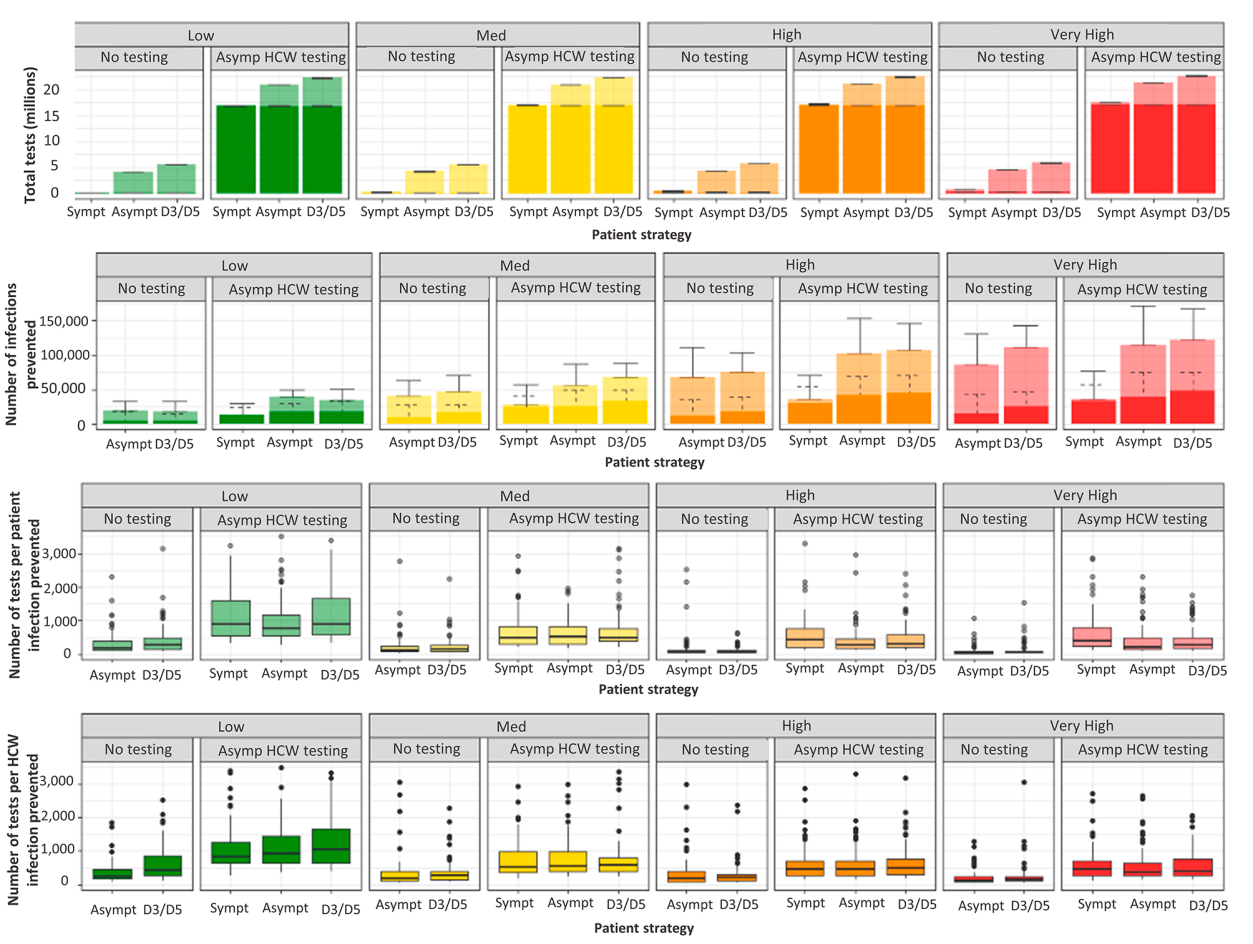
Efficiency of asymptomatic testing strategies. (**A**) Total tests required over 12-week simulation period for patients (light) and HCWs (dark). (**B**) Number of patient (light) and HCW (dark) infection prevented under each strategy compared to symptomatic testing only. (**C**) Number of tests required to prevent a single patient infection compared to symptomatic testing only. (**D**) Number of tests required to prevent a single HCW infection compared to symptomatic testing only

The number of tests required over the 12-week time period ranged from a minimum of 185,000 in the lowest prevalence scenario when only symptomatic patients and HCWs were tested to 22.5 million when HCWs are tested twice weekly, and patients are tested on admission and again at days 3 and 5–7 post admission in the highest prevalence setting (Fig. 4). The number of patient tests ranged from 70,000 in a low prevalence scenario with no asymptomatic testing to 5.5 million in a very high prevalence setting when asymptomatic patients are tested on admission and again at days 3 and 5–7 post-admission. The number of HCW tests required ranges from 10,000 when only symptomatic HCWs are tested in a low prevalence setting to 17 million when asymptomatic HCWs are tested twice weekly. When included in a strategy, testing asymptomatic HCWs twice weekly testing adds 16.9 million tests over the entire time period (two per week for 700,000 people).

The number of infections prevented by asymptomatic testing increases with the population prevalence. The number of patient infections prevented ranges from 14,000 (4,000–23,00) in a low prevalence scenario to 76,000 (60,000–95,000) in the highest prevalence scenario. The number of HCW infections prevented by asymptomatic testing ranges from 7000 (3000–14,000) in a low prevalence scenario to 21,350 (12,000–36,000) in the highest prevalence scenario (Fig. 4).

The minimum number of tests required to prevent a single patient infection occurs in the very high prevalence scenario when asymptomatic patients are tested on admission and only new symptomatic patients are tested during their stay (60, 50–80). However, in every prevalence scenario the most efficient patient testing strategy (in terms of tests required to prevent a single patient infection) was testing symptomatic patients only, followed by testing all patients on admission and retesting patients at day 3 and days 5–7 post-admission. The least efficient strategy for preventing patient infections across all prevalences was repeat testing of patients combined with twice weekly asymptomatic HCW testing. This strategy was least efficient in a low prevalence scenario, with 1,000 (700–2,000) tests required to prevent a single patient infection.

For HCWs, in scenarios where asymptomatic HCWs were tested twice weekly the number of tests to prevent a single HCW infection ranged from 850 (550–1500) in a very high prevalence scenario to 2500 (1350–4750) in a low prevalence scenario (Fig. 4). Despite preventing very few HCW infections, testing all patients on admission is the most efficient way to prevent HCW infections due to the small number of tests required compared to HCW testing strategies. Testing patients on admission requires 200 (425–800) tests to prevent a HCW infection.

### Sensitivity analysis

**Fig. 5:**
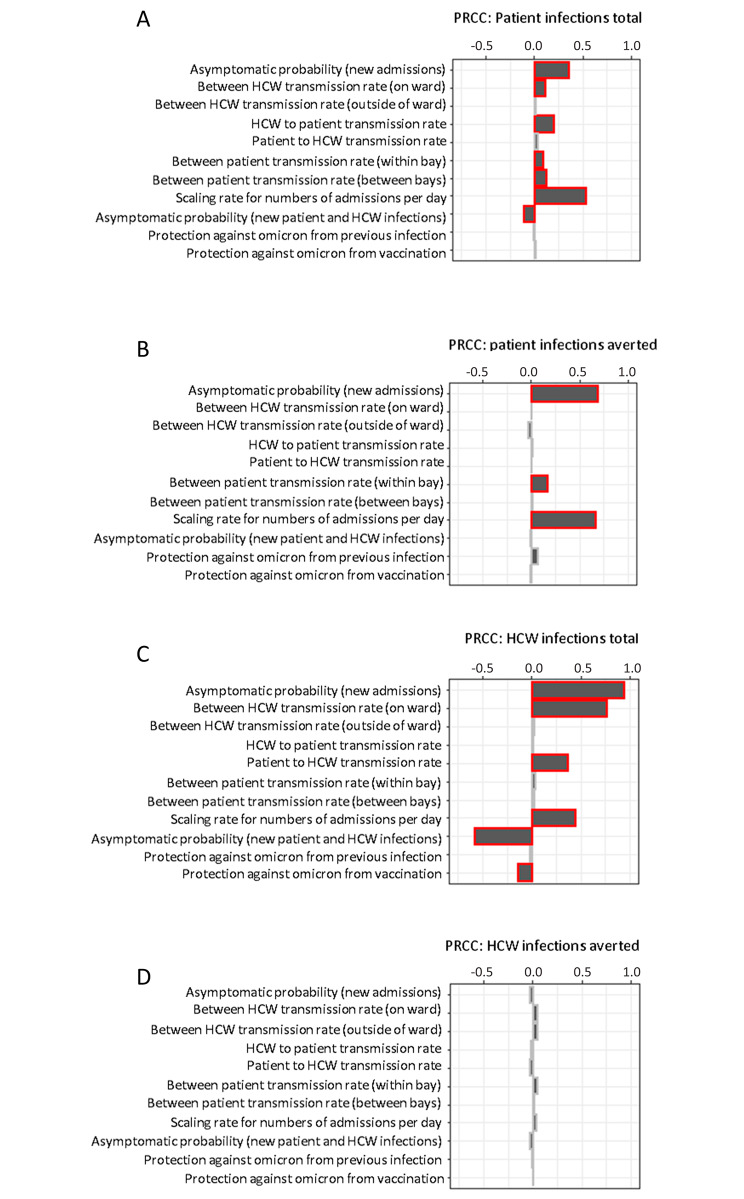
Sensitivity analysis. Partial-rank correlation coefficients were calculated for the number of nosocomial patient infections in the absence of asymptomatic testing (**A**), number of nosocomial patient infections averted when asymptomatic testing of patient and HCWs was on vs. off (**B**), total number of HCW infections in the absence of asymptomatic testing (**C**), and total number of HCW infections averted when asymptomatic testing of patient and HCWs was on vs. off (**D**). Simulations were carried out for a 12 week omicron-like time period of medium prevalence

A sensitivity analysis was performed on both the number of patient and HCW infections in total (in a scenario where symptomatic patients and HCWs were tested), and the number of infections averted by asymptomatic testing of patients and HCWs (compared to a baseline of symptomatic testing), over a 12-week medium prevalence time period (Fig. 5).

Positive partial-rank correlation coefficients were calculated (PRCCs) between the number of nosocomial patient cases and both the number of infected admissions per day and the probability a new admission is asymptomatic (i.e. the probability an admission is in hospital *for* COVID-19 vs. *with* COVID-19). This suggests that the number of nosocomial patient infections would be higher in a scenario where there was an increased number of infected patients admitted or if there was an increase in the proportion of admitted patient cases that were missed and therefore incorrectly cohorted on admission (Fig. 5A). Smaller, but still positive PRCCs were also calculated for all “to patient” transmission probabilities, with increasing the HCW to patient transmission rate having a greater effect than increasing the transmission probability within bays or wards. The PRCC for the probability that a new infection is asymptomatic was negative, meaning that if a higher proportion of cases became asymptomatic fewer nosocomial patient infections would occur (Fig. 5A). This is due to the dominance of the patient-to-patient transmission route and the shorter length of stay (and therefore exposure time) of asymptomatic patients compared to those that develop symptoms.

In addition, asymptomatic testing could avert a higher number of patient infections if the proportion of new admissions that were infected with SARS-CoV-2 was increased, the probability an infected new admission is asymptomatic is higher, or there is an increased risk of transmission within a bay (Fig. 5B).

For HCWs, positive PRCCs were found between the total number of infections and the probability an infected new patient admission is asymptomatic on admission, the between HCW transmission rates, and the scaling factor for the number of infected admissions (Fig. 5C). The probability a new infection is asymptomatic and the level of protection against Omicron vaccination have negative PRCCs. None of the varied parameters had significant PRCCs with the number of infections averted in HCWs (Fig. 5D).

## Discussion

This study modelled the impact of asymptomatic SARS-CoV-2 testing of patients and HCWs for preventing nosocomial transmission during hypothetical periods of low (0.5-1%), medium (1–2%), high (2–4%) and very high (4–8%) community prevalence of the omicron variant of the virus. We assume that 50% of HCWs and 30% of patients had some protection from previous infection with a non-Omicron SARS-CoV-2 variant and that 90% of HCWs and 85% of patients over 50 had received a booster vaccine and were therefore less likely to be infected with Omicron. Asymptomatic SARS-CoV-2 testing of patients and HCWs requires up to 22.5 million LFD tests over a 12-week period when patients are tested on admission, at day 3 and between days 5–7 post-admission and HCWs are tested twice weekly. When the community prevalence is higher than 4%, up to 62,500 patients and 48,300 HCW infections are prevented over a 12-week period by the most stringent asymptomatic testing policy. We found that testing HCW asymptomatically did not significantly affect the proportion of patients developing an infection in any scenario, but that testing all patients on admission reduced nosocomial infections in the patient population by up to 21.5%. Our modelling results did not find any significant benefit of repeat testing at days 3 and 5 post admission for reducing the risk of nosocomial infections in hospital inpatients. Conversely, we found that asymptomatic testing of HCWs had a moderate effect on the proportion of HCWs absent per month, but asymptomatic patient testing had only a small impact on absences or infections in the HCW population. There was no significant operational benefit in terms of either bed occupancy or HCW absence rates when either patients or HCWs were tested asymptomatically compared to symptomatic testing only.

Testing asymptomatic patients on admission was the most efficient strategy for preventing nosocomial infections of all of the asymptomatic testing strategies considered (compared to symptomatic testing only) and was most effective in a scenario where the population prevalence was very high. However, symptomatic testing only was more efficient in terms of number of cases detected per test. Testing asymptomatic HCWs twice weekly prevented a small proportion of HCW infections but was very inefficient with a minimum of 850 tests required to prevent a single HCW infection. The limited efficacy of asymptomatic HCW testing for preventing staff absences is reconcilable with results from other studies that have demonstrated that a large proportion of HCW infections are community-associated [2, 30], and those demonstrating that nosocomial transmission is most common between patients or between HCWs with transmission between patients and HCWs being significantly less common [2, 31, 32]. Further, we have previously demonstrated that strategies decreasing nosocomial transmission rates in HCWs often result in some level of increase in the number of HCWs that acquire an infection in the community [33]. We simulated a period with wide-spread community transmission. If the transmission rate in the community was small then HCW testing becomes much more impactful and this has been demonstrated in a modelling study over the first wave when a higher proportion of infections were healthcare associated [25, 34]. In addition, HCWs may be largely protected by policies around universal masking in communal areas and when treating patients [33], although the true efficacy of face masks for infection prevention is not known and varies between studies [35, 36].

Many model parameters were highly uncertain at the height of the pandemic, and sensitivity analyses allow judgment to be made around the risk associated with implementing different testing strategies. Our results suggest that when implemented in addition to symptomatic testing of patients and HCWs, twice-weekly asymptomatic testing of HCWs is unlikely to be an effective strategy for infection prevention over a similar time period (i.e. where there is widespread community transmission), regardless of how transmissible a SARS-CoV-2 variant is, the impact of IPC methods that are in place to reduce the transmission rate [33], or how many patients are admitted to hospital.

Sensitivity analysis results also indicate that asymptomatic patient testing could significantly reduce the number of nosocomial patient infections if a new variant emerges where the probability of an infection being asymptomatic is higher. In practice, the removal of asymptomatic testing in hospitals and the community means that the asymptomatic rate of a new variant may not be unknown, unless cohort studies such as the SARS-CoV-2 immunity and reinfection evaluation (SIREN) study continues [4] or the Office of National Statistics (ONS) COVID-19 infection survey is restarted, making it difficult to determine whether restarting asymptomatic testing would be beneficial. However, we would expect the health impact of increased transmission associated with a higher asymptomatic probability in the absence of asymptomatic testing to be small, as new infections would also have a higher likelihood of being asymptomatic and would therefore be less likely to require interventions or experience clinical symptoms. There would therefore be little benefit of restarting asymptomatic testing in this scenario providing some assurance that removing asymptomatic testing would not significantly impact health outcomes. Another factor that significantly increased the efficacy of asymptomatic testing was increasing the number of SARS-CoV-2 positive admissions. This measure is observable under a strategy of symptomatic testing only and therefore could be used as a trigger to restart asymptomatic patient testing for infection prevention if required. Finally, sensitivity analyses suggest that asymptomatic patient testing could be more impactful in scenarios where the rate of transmission between patients in the same bay is higher, e.g. in settings with poor ventilation, where bays where beds have to be closer together, or if a more transmissible variant emerges. The transmissibility of a virus in a particular setting is difficult to ascertain without asymptomatic testing and epidemiological or genomic evaluation, however the association between the within-bay transmission rate and the efficacy of asymptomatic testing for infection prevention is weak, suggesting a very large change in transmission rates would be required for asymptomatic patient testing to have a significant impact. Conversely if the rate of asymptomatic infection decreased over time, asymptomatic testing would prevent a smaller number of transmission events but could have health benefits if patients at risk of developing severe disease were offered treatment prior to becoming symptomatic. In this work we do not consider the impact of symptomatic testing. In another modelling study we demonstrated that removing symptomatic patient testing could have caused up to a 35% increase in the number of patients nosocomially infected over the entire pandemic [33]. Studies suggest that asymptomatically infected individuals are less infectious than those who are symptomatically infected [22, 37], however even if transmissibility of asymptomatic cases increased the short length of stay of non-COVID-19 patients in hospital means that asymptomatic screening on days 3 and 5–7 does not significantly increase detection rates of asymptomatic nosocomially-infected individuals as many are discharged before becoming detectable [38]. It has also previously been shown that patients with nosocomial infections, rather than those with community-acquired infections, are the main source of patient-to-patient transmission, therefore it is unlikely that testing asymptomatic patients on admission would have a large effect even if infectiousness of asymptomatic patients was the same as symptomatic. Counterfactual mathematical modelling is an important tool for evaluating the impact of interventions and provides rapid output in the absence of large-scale trials. The model used in this work has been continuously curated throughout the pandemic and has been calibrated to the best available data on HCW and patient infections. The results presented represent an upper bound on efficacy of asymptomatic testing, and therefore are an upper-bound on how efficient asymptomatic testing strategies are. As population immunity to Omicron builds or repeat rounds of vaccination are introduced the efficiency of testing strategies other than the baseline (symptomatic testing of patients and HCWs only) would become less so. While this is not an economic study, these outputs can be useful in resource allocation decision making and providing insight into prioritisation of strategies, given the opportunity cost of spending on less efficient testing strategies. Future work following through the patient health and NHS monetary costs of the scenarios presented here will be key in understanding this opportunity cost in standard “cost per quality-adjusted life year gained” and “incremental cost-effectiveness ratio” metrics and would need to include the impact of infections on disease prevention, hospitalisation, and mortality.

The results generated with this complex IBM are supported by those obtained using previously published ordinary differential equation (ODE) model [34]. This simpler modelling approach was used to demonstrate the impact of regular testing early in the pandemic before LFD tests were available and results suggested that regular (7-day) testing prevented an average of one nosocomial transmission event per day, and therefore in practice would have limited efficacy for preventing staff absences in a time period where nosocomial transmission was responsible for a large proportion of infections in HCWs. This aligns with the IBM results that also found limited effectiveness of repeated HCW testing for preventing HCW infections.

There are several limitations to this study. We present estimates of the impact of removing asymptomatic testing using a model that has been developed through consultation with clinical experts, however like all models ours is an abstraction of a real-world process. We report total numbers of SARS-CoV-2 infections in HCWs and total nosocomial infections in patients, but in practice ascertainment rate is determined by testing policy. Other modelling studies have suggested that only 30% of infections that occur in patients are observed in hospitals [25], and following the removal of asymptomatic testing we would expect all asymptomatic HCW infections to be missed (~ 40% [5]). We do not discriminate between types of admissions, and therefore cannot provide guidance on scenarios where testing should be continued. It is assumed that compliance to testing is constant over the simulation period and that adherence to testing is “all-or-nothing”, so asymptomatic HCWs either test twice weekly or do not test at all over the entire time period. In practice adherence to testing likely changes with prevalence. In this model we do not consider admission diagnosis when calculating length of stay and therefore do not capture additional benefits of detecting and treating infections in patients with complications such as dementia, cardiac disease, or cancer [39]. Further, we do not explore the impact of staff testing by ward types, although another study using this model explores the efficacy of testing ward-based staff using a pooled-testing framework [40].

## Conclusions

Modelling suggests that when vaccination uptake or protection from previous infection is high asymptomatic testing of patients and HCWs in an Omicron-like setting has a small effect on the number of patients and HCWs infected with SARS-CoV-2 over a 12-week period compared to symptomatic testing alone. Testing all patients on admission is the most efficient strategy for preventing patient and HCW infections compared to a baseline strategy of symptomatic testing only, but symptomatic testing only is the most efficient strategy in terms of tests per case detected. No additional benefit was seen from additional patient testing at days 3 and 5 post-admission compared to testing all patients on admission only, under the assumption that symptomatic testing remains. Strategies involving HCW testing are highly inefficient for infection prevention in HCWs due to the large number of tests require and the risk of HCWs developing an infection in the community. If healthcare-associated transmission became a dominant source of infection (e.g. during times when interventions such as lockdowns are in place to reduce community transmission) the impact of asymptomatic testing could be greater. This study found that the impact of asymptomatic testing for infection prevention is small However if healthcare-associated transmission became a dominant source of infection (e.g. during times when interventions such as lockdowns are in place to reduce community transmission) the impact of asymptomatic testing could be greater A full health economic evaluation should be performed to determine the health-related impact of reducing SARS-CoV-2 infections in patients and HCWs. We found that the efficacy and fun efficiency of testing policies varied between different prevalence levels and policy makers may want to consider changing testing guidance with prevalence. There are however many operational challenges in rapidly changing testing guidance that would make this difficult in practice. A possible approach would be to have seasonal guidance with more intensive testing recommended in times of higher prevalence (e.g. Winter months), and a more relaxed approach over Summer.

### Electronic supplementary material

Below is the link to the electronic supplementary material.

Supplementary Material 1

Supplementary Material 2

Supplementary Material 3

Supplementary Material 4

Supplementary Material 5

Supplementary Material 6

Supplementary Material 7

## Data Availability

Simulation data available on request from Stephanie Evans (stephanie.evans@ukhsa.gov.uk).

## Abbreviations

SARS-CoV-2: Severe acute respiratory virus 2
HCW: Healthcare worker
IBM: Individual-based model
PRCCs: Partial-rank correlation coefficient

## Declarations

### Patient and public involvement

Patients were not involved in this study.
