# Supplementary material for: The effectiveness and efficiency of asymptomatic SARS-CoV-2 testing strategies for patient and healthcare workers within acute NHS hospitals during an omicron-like period
Source: BMC Infect Dis. 2024 Jan 8;24:64. doi: 10.1186/s12879-023-08948-9 (PMC10775431; doi:10.1186/s12879-023-08948-9)
Supplement: Supplementary file 1 — Supplementary Material 1 [file 12879_2023_8948_MOESM1_ESM.docx]

**Table S1: Ranges and parameters used in sensitivity analysis**

| Parameter |  | Min | Max |
| --- | --- | --- | --- |
| admAsymProb | Probability a new infected admission will be asymptomatic | 0.101 | 0.8 |
| bP2P | Transmission rate between patients in a bay | 0.00631 | 0.00915 |
| bP2P_hosp | Transmission rate between patients in a ward | 0.00013 | 0.000186 |
| bH2P | Transmission rate from HCWs to patients | 0.0004 | 0.000997 |
| newInfAsymProb | Probability a new infection will be asymptomatic | 0.101 | 0.798 |
| bP2H | Transmission rate from patients to HCWs | 0.0004 | 0.000997 |
| bH2H | Transmission rate between HCWs | 0.0001 | 0.000175 |
| commScale | Rate of HCW infection in the community | 0.204 | 0.314 |
| vaccine_efficacy | Vaccine induced protection rate | 0.00023 | 0. 7 |
| caseScale | Scaling factor for admissions | 0.252 | 2 |
