# Supplementary material for: The effectiveness and efficiency of asymptomatic SARS-CoV-2 testing strategies for patient and healthcare workers within acute NHS hospitals during an omicron-like period
Source: BMC Infect Dis. 2024 Jan 8;24:64. doi: 10.1186/s12879-023-08948-9 (PMC10775431; doi:10.1186/s12879-023-08948-9)
Supplement: Supplementary file 2 — Supplementary Material 2 [file 12879_2023_8948_MOESM2_ESM.docx]

**Table S2: Infection rates in patients and HCWs under the scenarios included in the study**

| Testing strategy | | Prevalence | % HCW infected (total) | | Number of HCW infected (total, Nht) | | % HCW infected (nosocomial) | | Number of HCW infected (nosocomial, Nh) | | % patients infected (nosocomial) | | Number of patients infected (nosocomial, Np) | |
| --- | --- | --- | --- | --- | --- | --- | --- | --- | --- | --- | --- | --- | --- | --- |
| d5+d3 | No HCW testing | Low | 6 | (4, 8.8) | 42,100 | (27,900, 61,300) | 3.9 | (2.5, 6) | 27,300 | (17,200, 42,300) | 3.4 | (3.2, 3.6) | 139,000 | (131,000, 147,000) |
| d5+d3 | No HCW testing | Med | 11.8 | (7.5, 15.6) | 82,800 | (52,300, 109,000) | 7.5 | (4.4, 10.9) | 52,400 | (30,900, 76,400) | 4.2 | (3.9, 4.6) | 170,000 | (158,000, 184,000) |
| d5+d3 | No HCW testing | High | 20 | (13.6, 28) | 140,000 | (95,500, 196,000) | 12.3 | (8.2, 18) | 86,100 | (57,400, 126,000) | 5.8 | (5.1, 6.5) | 235,000 | (208,000, 263,000) |
| d5+d3 | No HCW testing | Very High | 33.2 | (22.7, 43.6) | 232,000 | (159,000, 305,000) | 18.8 | (13, 26.1) | 132,000 | (90,900, 183,000) | 8.1 | (7.3, 8.9) | 329,000 | (295,000, 360,000) |
| d5+d3 | Asymp HCW testing | Low | 5.2 | (3.5, 7.1) | 36,200 | (24,800, 49,600) | 3 | (2, 4.4) | 20,900 | (14,200, 30,700) | 3.4 | (3.2, 3.6) | 136,000 | (128,000, 144,000) |
| d5+d3 | Asymp HCW testing | Med | 10.2 | (7.3, 13.5) | 71,600 | (50,900, 94,600) | 5.8 | (4, 8.4) | 40,600 | (28,400, 59,100) | 4.2 | (3.8, 4.5) | 169,000 | (155,000, 183,000) |
| d5+d3 | Asmymp HCW testing | High | 19.1 | (12.7, 24.1) | 133,000 | (88,600, 169,000) | 10.4 | (6.9, 15) | 73,100 | (48,500, 105,000) | 5.7 | (5.1, 6.3) | 230,000 | (204,000, 255,000) |
| d5+d3 | Asymp HCW testing | Very High | 30.9 | (22.2, 40.3) | 216,000 | (155,000, 282,000) | 16.4 | (11.4, 22.1) | 115,000 | (80,000, 155,000) | 8 | (7, 8.8) | 323,000 | (284,000, 354,000) |
| Adm | No HCW testing | Low | 6.5 | (4, 8.9) | 45,400 | (28,100, 62,000) | 4 | (2.6, 6.1) | 27,900 | (17,900, 42,800) | 3.4 | (3.2, 3.6) | 138,000 | (131,000, 147,000) |
| Adm | No HCW testing | Med | 12 | (7.7, 15.8) | 84,000 | (53,700, 111,000) | 7.8 | (4.8, 11) | 54,800 | (33,300, 77,000) | 4.1 | (3.8, 4.6) | 168,000 | (154,000, 184,000) |
| Adm | No HCW testing | High | 20.9 | (13.9, 28.2) | 147,000 | (97,600, 197,000) | 12.9 | (8.4, 18.2) | 90,100 | (58,500, 128,000) | 5.7 | (5.1, 6.3) | 232,000 | (206,000, 253,000) |
| Adm | No HCW testing | Very High | 33.2 | (23.5, 44.3) | 233,000 | (164,000, 310,000) | 19.2 | (13.9, 27.4) | 135,000 | (97,400, 192,000) | 8.3 | (7.3, 8.9) | 334,000 | (297,000, 360,000) |
| Adm | Asymp HCW | Low | 5.4 | (3.6, 7.2) | 38,100 | (25,200, 50,500) | 3.1 | (2, 4.4) | 21,800 | (14,300, 31,100) | 3.4 | (3.2, 3.6) | 136,000 | (127,000, 144,000) |
| Adm | Asymp HCW testing | Med | 10.4 | (7.1, 13.9) | 72,500 | (49,500, 97,300) | 6 | (4.2, 8.6) | 42,100 | (29,200, 59,900) | 4.1 | (3.8, 4.5) | 167,000 | (152,000, 181,000) |
| Adm | Asymp HCW testing | High | 17.9 | (12.4, 23.7) | 125,000 | (87,200, 166,000) | 10.1 | (7.3, 14.6) | 70,600 | (50,900, 102,000) | 5.5 | (4.9, 6.2) | 222,000 | (199,000, 249,000) |
| Adm | Asympt HCW testing | Very High | 31.1 | (22, 40.1) | 218,000 | (154,000, 280,000) | 16.3 | (11.3, 23.6) | 114,000 | (79,300, 165,000) | 7.9 | (6.9, 8.7) | 319,000 | (280,000, 353,000) |
| Sympt | No HCW testing | Low | 6.3 | (4, 9.2) | 43,800 | (28,100, 64,000) | 4.1 | (2.4, 6.2) | 28,500 | (17,000, 43,500) | 3.7 | (3.4, 4.1) | 149,000 | (139,000, 165,000) |
| Sympt | No HCW testing | Med | 12 | (8.2, 17.3) | 84,000 | (57,800, 121,000) | 8.1 | (5.1, 12.1) | 56,500 | (35,700, 84,600) | 4.9 | (4.3, 5.6) | 197,000 | (175,000, 225,000) |
| Sympt | No HCW testing | High | 21.2 | (14.4, 29.5) | 149,000 | (101,000, 207,000) | 13.8 | (8.8, 20.2) | 96,300 | (61,600, 141,000) | 7 | (6.1, 8) | 284,000 | (247,000, 324,000) |
| Sympt | No HCW testing | Very High | 35.3 | (23.5, 46.3) | 247,000 | (164,000, 324,000) | 21.3 | (14.3, 30.2) | 149,000 | (100,000, 211,000) | 9.9 | (8.8, 10.9) | 399,000 | (355,000, 442,000) |
| Sympt | Asymp HCW testing | Low | 5.2 | (3.8, 7.7) | 36,700 | (26,800, 54,100) | 3.1 | (2.2, 4.9) | 22,000 | (15,600, 34,500) | 3.7 | (3.5, 4) | 150,000 | (140,000, 160,000) |
| Sympt | Asymp HCW testing | Med | 10.9 | (7.1, 14.1) | 76,600 | (49,400, 98,900) | 6.4 | (4.2, 9.1) | 44,600 | (29,500, 63,700) | 4.8 | (4.3, 5.4) | 194,000 | (172,000, 220,000) |
| Sympt | Asymp HCW Testing | High | 19.3 | (13.6, 25.5) | 135,000 | (94,900, 179,000) | 11.1 | (7.7, 15.7) | 77,700 | (53,900, 110,000) | 7 | (5.8, 7.9) | 282,000 | (236,000, 321,000) |
| Sympt | Asymp HCW testing | Very High | 31.4 | (23.3, 42.2) | 220,000 | (163,000, 296,000) | 17.3 | (12.7, 25.1) | 121,000 | (88,800, 176,000) | 9.7 | (8.6, 10.9) | 393,000 | (347,000, 441,000) |
