# Supplementary material for: The effectiveness and efficiency of asymptomatic SARS-CoV-2 testing strategies for patient and healthcare workers within acute NHS hospitals during an omicron-like period
Source: BMC Infect Dis. 2024 Jan 8;24:64. doi: 10.1186/s12879-023-08948-9 (PMC10775431; doi:10.1186/s12879-023-08948-9)
Supplement: Supplementary file 3 — Supplementary Material 3 [file 12879_2023_8948_MOESM3_ESM.docx]

**Table S3: Number of infections and cost per case averted compared to no asymptomatic patient or HCW testing**

| Prevalence | Patient testing scenario | HCW testing scenario | Testing cost  (weekly) | | Patient infections prevented (total) | | Testing cost per patient infection averted | | HCW infections  prevented (tot) | | Testing cost per HCW infection prevented | |
| --- | --- | --- | --- | --- | --- | --- | --- | --- | --- | --- | --- | --- |
| Low | d5+d3 | No testing | 830,000 | (830000, 830000) | 13,000 | (4300, 23000) | 290 | (170, 530) | 2,500 | (-350, 5500) | 920 | (-620, 1900) |
| Low | d5+d3 | Asymp HCW testing | 3,500,000 | (3500000, 3500000) | 14,000 | (4000, 23000) | 1,200 | (720, 2000) | 8,100 | (4100, 15000) | 2,400 | (1400, 4800) |
| Low | Adm | No testing | 630,000 | (630000, 630000) | 15,000 | (5300, 24000) | 210 | (130, 350) | 1,600 | (-1200, 4800) | 510 | (-980, 1200) |
| Low | Adm | Asymp HCW testing | 3,300,000 | (3300000, 3300000) | 17,000 | (8900, 27000) | 1,100 | (710, 1900) | 8,200 | (4200, 14000) | 2,200 | (1300, 4500) |
| Low | Sympt | Asymp HCW testing | 2,600,000 | (2600000, 2600000) | 1,500 | (-7300, 8200) | 880 | (-1900, 2400) | 5,800 | (2200, 13000) | 2,100 | (1100, 5000) |
| Med | d5+d3 | No testing | 830,000 | (830000, 830000) | 27,000 | (15000, 38000) | 180 | (130, 300) | 3,700 | (-44, 8600) | 620 | (-95, 1400) |
| Med | d5+d3 | Asymp HCW testing | 3,500,000 | (3500000, 3500000) | 28,000 | (19000, 39000) | 750 | (550, 1100) | 14,000 | (6400, 22000) | 1,500 | (840, 3000) |
| Med | Adm | No testing | 620,000 | (620000, 620000) | 30,000 | (17000, 41000) | 130 | (92, 210) | 2,500 | (-1100, 6000) | 480 | (-740, 1100) |
| Med | Adm | Asymp HCW testing | 3,300,000 | (3300000, 3300000) | 32,000 | (20000, 46000) | 630 | (440, 1000) | 13,000 | (6300, 22000) | 1,500 | (830, 2700) |
| Med | Sympt | Asymp HCW testing | 2,600,000 | (2600000, 2600000) | 2,000 | (-7100, 10000) | 740 | (-1800, 2000) | 9,900 | (3800, 19000) | 1,500 | (770, 3300) |
| High | d5+d3 | No testing | 820,000 | (820000, 820000) | 49,000 | (34000, 63000) | 110 | (82, 150) | 7,600 | (2300, 13000) | 490 | (280, 950) |
| High | d5+d3 | Asymp HCW testing | 3,400,000 | (3400000, 3500000) | 52,000 | (39000, 68000) | 420 | (320, 560) | 22,000 | (11000, 34000) | 1,000 | (630, 1800) |
| High | Adm | No testing | 610,000 | (610000, 610000) | 51,000 | (36000, 66000) | 76 | (59, 110) | 6,200 | (1100, 12000) | 380 | (210, 720) |
| High | Adm | Asymp HCW testing | 3,200,000 | (3200000, 3200000) | 54,000 | (40000, 71000) | 380 | (290, 520) | 19,000 | (10000, 34000) | 1,100 | (610, 1900) |
| High | Sympt | Asymp HCW testing | 2,600,000 | (2600000, 2600000) | 4,300 | (-5800, 13000) | 770 | (-1400, 1800) | 15,000 | (7600, 27000) | 1,100 | (590, 2000) |
| Very High | d5+d3 | No testing | 800,000 | (800000, 800000) | 70,000 | (54000, 84000) | 73 | (61, 95) | 12,000 | (6300, 19000) | 390 | (250, 750) |
| Very High | d5+d3 | Asymp HCW testing | 3,400,000 | (3400000, 3400000) | 76,000 | (59000, 91000) | 290 | (240, 370) | 25,000 | (15000, 41000) | 860 | (530, 1500) |
| Very High | Adm | No testing | 600,000 | (590000, 600000) | 75,000 | (57000, 91000) | 51 | (42, 67) | 7,900 | (3300, 15000) | 360 | (210, 680) |
| Very High | Adm | Asymp HCW testing | 3,200,000 | (3200000, 3200000) | 80,000 | (63000, 95000) | 260 | (220, 330) | 24,000 | (13000, 37000) | 850 | (540, 1500) |
| Very High | Sympt | Asymp HCW testing | 2,600,000 | (2600000, 2600000) | 6,500 | (-2800, 16000) | 810 | (-1100, 1800) | 17,000 | (9100, 29000) | 970 | (560, 1700) |
