# Supplementary figures and images for: The effectiveness and efficiency of asymptomatic SARS-CoV-2 testing strategies for patient and healthcare workers within acute NHS hospitals during an omicron-like period
Source: BMC Infect Dis. 2024 Jan 8;24:64. doi: 10.1186/s12879-023-08948-9 (PMC10775431; doi:10.1186/s12879-023-08948-9)

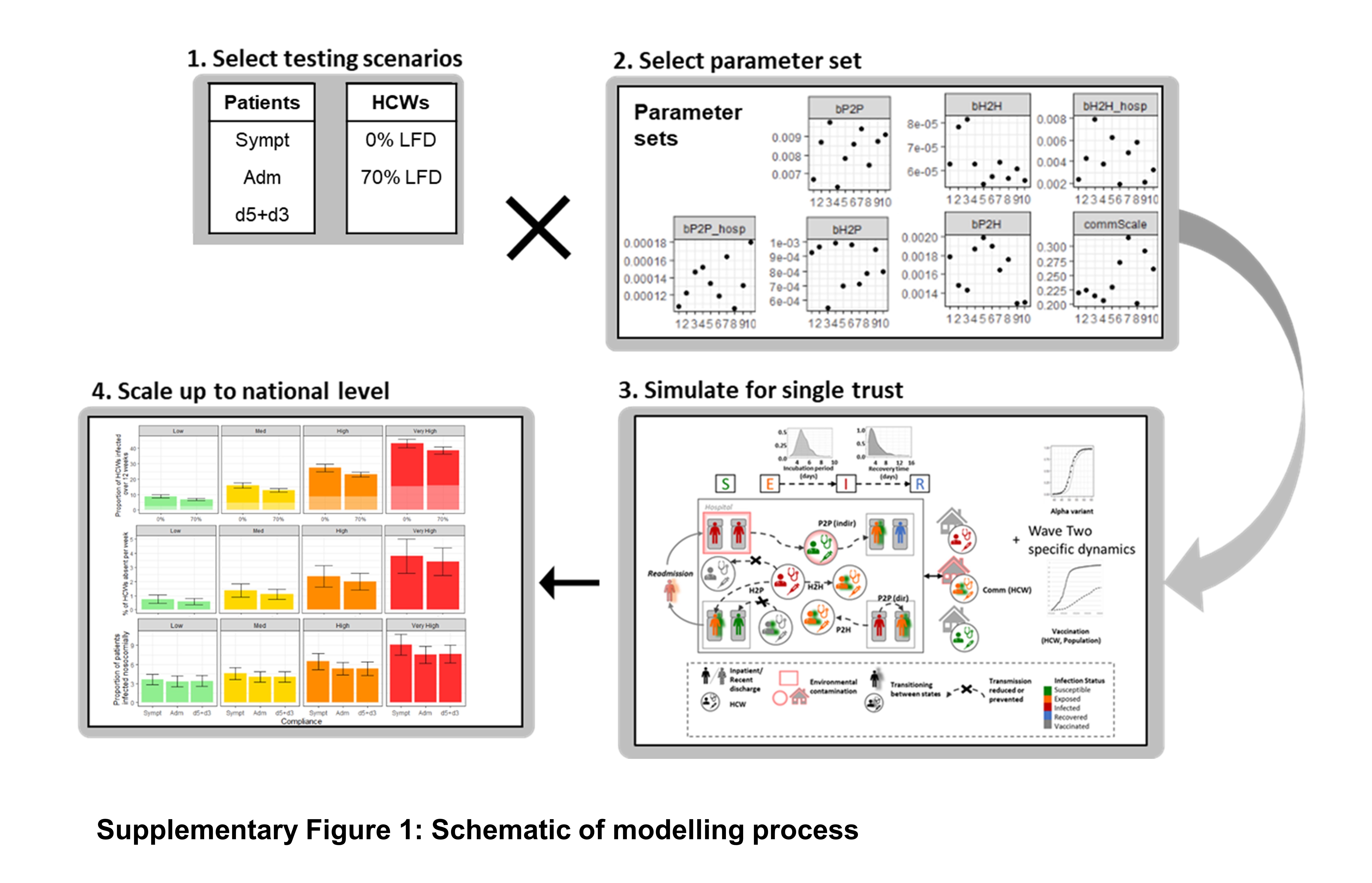

Supplement: Supplementary file 4 — Supplementary Material 4 [file 12879_2023_8948_MOESM4_ESM.jpg]

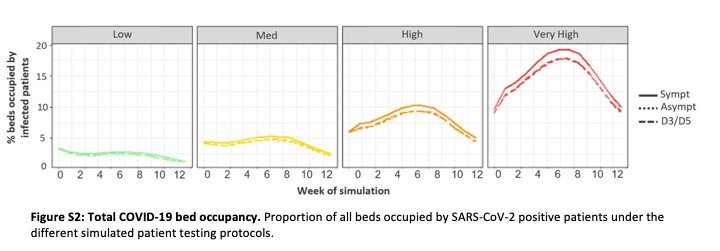

Supplement: Supplementary file 5 — Supplementary Material 5 [file 12879_2023_8948_MOESM5_ESM.jpg]

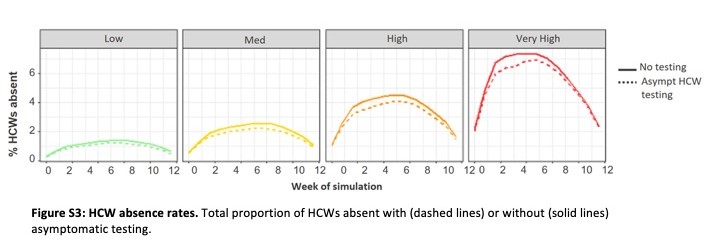

Supplement: Supplementary file 6 — Supplementary Material 6 [file 12879_2023_8948_MOESM6_ESM.jpg]
